# Prospective methods for identifying perioperative risk‐assessment methods for patient safety over 20 years: a systematic review

**DOI:** 10.1002/bjs5.50246

**Published:** 2019-12-17

**Authors:** A. J. Heideveld‐Chevalking, H. Calsbeek, J. Hofland, W. J. H. J. Meijerink, A. P. Wolff

**Affiliations:** ^1^ Department of Operating Rooms Radboud University Medical Centre Nijmegen the Netherlands; ^2^ IQ healthcare, Radboud Institute for Health Sciences Radboud University Medical Centre Nijmegen the Netherlands; ^3^ Department of Anaesthesiology Radboud University Medical Centre Nijmegen the Netherlands; ^4^ Department of Anaesthesiology University of Groningen Medical Centre Groningen the Netherlands

## Abstract

**Background:**

Serious preventable surgical events still occur despite considerable efforts to improve patient safety. In addition to learning from retrospective analyses, prospective risk‐assessment methods may help to decrease preventable events further by targeting perioperative hazards. The aim of this systematic review was to assess the methods used to identify perioperative patient safety risks prospectively, and to describe the risk areas targeted, the quality characteristics and feasibility of methods.

**Methods:**

MEDLINE, Embase, CINAHL and Cochrane databases were searched, adhering to PRISMA guidelines. All studies describing the development and results of prospective methods to identify perioperative patient safety risks were included and assessed on methodological quality. Exclusion criteria were interventional studies, studies targeting one specific issue, studies reporting on structural factors relating to fundamental hospital items, and non‐original or case studies.

**Results:**

The electronic search resulted in 16 708 publications, but only 20 were included for final analysis, describing five prospective risk‐assessment methods. Direct observation was used in most studies, often in combination. Direct (16 studies) and indirect (4 studies) observations identified (potential) adverse events (P)AEs, process flow disruptions, poor protocol compliance and poor practice performance. (Modified) Healthcare Failure Mode and Effect Analysis (HFMEA™) (5 studies) targeted potential process flow disruption failures, and direct (P)AE surveillance (3 studies) identified (P)AEs prospectively. Questionnaires (3 studies) identified poor protocol compliance, surgical flow disturbances and patients' willingness to ask questions about their care. Overall, quality characteristics and feasibility of the methods were poorly reported.

**Conclusion:**

The direct (in‐person) observation appears to be the primary prospective risk‐assessment method that currently may best help to target perioperative hazards. This is a reliable method and covers a broad spectrum of perioperative risk areas.

## Graphical abstract

Serious preventable surgical events continue to occur despite considerable efforts to improve patient safety. This systematic literature review provides an overview of prospective risk‐assessment methods used proactively to identify and target hazardous failures and preventable patient harm in perioperative patient care.

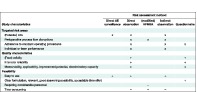

Patient safety risk factors

## Abstract (es)

**Antecedentes:**

A pesar de los esfuerzos considerables para mejorar la seguridad del paciente, aún se producen complicaciones quirúrgicas graves prevenibles. Además de adquirir conocimientos a través de los análisis retrospectivos, los métodos de evaluación de riesgos prospectivos por su enfoque en los riesgos perioperatorios, pueden ayudar a disminuir aún más los efectos adversos prevenibles. Esta revisión sistemática tiene como objetivo evaluar los métodos utilizados para identificar de forma prospectiva los riesgos perioperatorios de seguridad del paciente, describiendo las áreas de riesgo y las características de calidad y viabilidad de los métodos.

**Métodos:**

Se realizaron búsquedas en las bases de datos MEDLINE, EMBASE, CINAHL y Cochrane siguiendo las recomendaciones PRISMA. Se incluyeron todos los estudios que describían el desarrollo y los resultados de métodos prospectivos para identificar los riesgos perioperatorios de seguridad del paciente y se evaluó su calidad metodológica. Se excluyeron los estudios de intervención, aquellos estudios dirigidos a un tema específico, los estudios enfocados a factores estructurales relacionados con elementos hospitalarios clave, y los estudios no originales o series de casos.

**Resultados:**

La búsqueda electrónica identificó 16.708 publicaciones, pero solamente se incluyeron 20 publicaciones en las que se describían 5 métodos prospectivos de evaluación de riesgos. La observación directa fue el método utilizado en la mayoría de los estudios, a menudo en combinación con métodos indirectos. Las observaciones directas (80%) e indirectas (20%) identificaron efectos adversos potenciales (*potential adverse events*, (P)AEs), disrupciones en el flujo de los procesos, baja adherencia a los protocolos y prácticas deficientes. El análisis (modificado) de fallo de la atención sanitaria por modo y efecto (*Healthcare‐Failure‐Mode‐and‐Effect Analysis*) (25%) enfocado a fallos potenciales de disrupción de los procesos y la vigilancia directa de los (P)AEs (16%) identificaron (P)AEs de forma prospectiva. Los cuestionarios (15%) identificaron una baja adherencia a los protocolos, alteraciones en el flujo del proceso quirúrgico y la disposición de los pacientes para hacer preguntas sobre su atención. En general, las características de calidad y la viabilidad de los métodos se describían de manera deficiente.

**Conclusión:**

La observación directa (en persona) es el principal método prospectivo de evaluación de riesgos que actualmente podría ser el mejor método para el enfoque en los riesgos perioperatorios. Es un método confiable y cubre un amplio espectro de áreas de riesgo perioperatorio**.**

## Introduction

The surgical volume worldwide has been estimated at 312·9 million operations in 2012, an increase of 33·6 per cent over 8 years^1^. The surgical care pathway is complex, and serious adverse events (AEs) remain common^2^. An AE is usually defined as an unintended injury or complication resulting in prolonged hospital stay, disability at the time of discharge or death, caused by healthcare management rather than by the patient's underlying disease process^3^, ^4^, ^5^. Major studies^3^, ^4^, ^5^, ^6^, ^7^, ^8^, ^9^, ^10^, ^11^ have reported AE rates of 3–16 per cent and progress towards reduction seems lacking^12^. In addition, serious, potentially devastating, preventable surgical events, named ‘never events’, continue to occur despite considerable efforts to improve patient safety^2^, and are considered to be unacceptable^13^.

The incidence and estimates of wrong‐site surgery and retained surgical items in the US setting vary considerably by data source and procedure, with median estimates of one event per 100 000 and one per 10 000 surgical procedures respectively^14^. Wrong‐site surgery refers to surgery on the wrong side or at the wrong site, the wrong procedure, the wrong implant, or the wrong patient. Retained surgical items refers to items left unintentionally in a patient after surgery, some being clinically asymptomatic and even discovered a long time after the surgical procedure.

The AEs can also lead to severe consequences for clinicians and institutions, including the psychological effect on involved healthcare professionals, the financial burden of medicolegal action, and negative effects on a professional reputation. Further, patient harm generates a considerable strain on health system finances. Treating AEs might even contribute to about 15 per cent of hospital activity^15^. From an economic perspective, patient harm may cost trillions of dollars each year through loss of capacity and productivity of patients and their caregivers. In a political sense, the costs of safety failure include loss of trust in the health systems, governments, and social institutions^15^.

To apply the most efficient and effective interventions to decrease the AE rate in healthcare, assessments of safety risks must capture reliable information in dynamic and complex care situations. As a large proportion of AEs are related to the surgery, it has been advised^5^ that funds and efforts be concentrated on interventions aimed at reducing these types of event in this field.

Risk analysis is gaining significance to help organizations minimize risks of patient harm, and there is a growing need for better and systematic insight into methods available to perform such a prospective risk assessment. Prospective methods to measure patient risks have advantages over retrospective ones, as they do not have to rely on an AE having occurred and been reported, and allow for the identification of latent factors that may lead to hazards. In contrast to retrospective risk assessment^16^, little is known about the availability of prospective procedures. This study aimed to perform a systematic review of the literature on the prospective methods used to identify perioperative patient safety risks. This included the full perioperative path, from preoperative surgical and anaesthesia risk assessment to patient admission, surgical procedure and discharge from hospital. A secondary aim was to describe the kinds of risk area targeted per method and, if studied, to assess the quality characteristics and feasibility of each method.

## Methods

The methodology and reporting of this study was performed according to the PRISMA guidelines^17^. The types of included study and quality characteristics were categorized according to UK National Institute for Health and Care Excellence (NICE) public health guidelines^18^.

### Inclusion and exclusion criteria

All published literature in the English and Dutch language between 1 November 1999 and 23 May 2019, reporting primarily on methods assessing patient risks prospectively in a perioperative setting, was searched for inclusion. Original research papers were included if: they provided a clear description of methodology, population of interest, and results; and more than one single surgical subspecialty was involved in the studies (unless there was no doubt that the used method was applicable to other surgical specialties). Scientific publications were excluded if they met at least one of the following criteria: studies that described interventions on improvement of patient safety, such as implementation of the WHO Surgical Safety Checklist, or interventions on surgical team performance; studies in which only one specific patient safety issue was targeted, such as surgical‐site infection or medication safety; and studies reporting on structural factors relating to fundamental hospital items, such as staff qualifications and equipment skills. Narrative reviews, editorials, opinions, personal views, response letters, and case reports or case studies were also excluded.

### Information sources and searches

In May 2019, a search was performed using the following databases: MEDLINE (PubMed), Embase, the Cumulative Index to Nursing and Allied Health Literature (CINAHL) and the Cochrane Library. A full electronic search strategy for the MEDLINE database is presented in *Appendix* *S1* (supporting information). The studies were screened independently for eligibility on the basis of title and abstract; the full text was screened when the abstract was not available. Discrepancies resulting from article screening were discussed further to reach consensus; however, in cases of doubt, studies were still included. The full‐text content of selected publications was then screened for final inclusion or exclusion. Finally, all references of the included studies were searched manually to identify additional relevant studies.

### Study characteristics

For each selected study, the following key characteristics were extracted: period of study and country, aim of the study, study design (based on the NICE Appendix D Glossary of study designs^18^), perioperative phase, target group or sample size, and type of prospective measurement method.

### Study quality

The methodological quality was investigated using the NICE Appendix G Quality appraisal checklist^18^. Studies were excluded when graded a minus for overall internal or external validity.

### Risk‐assessment methods

For each reported method, the following data were extracted: a description of the method, the way of performing, identified risks and risk areas, and key conclusions. Feasibility and quality characteristics, such as measurability, applicability, discriminatory capacity and improvement potential, as well as validity characteristics were also extracted from publications if reported, using the grading or wording of the authors. Finally, an overview of employed methods and targeted risks was presented, and results were grouped and summarized.

## Results

**Figure 1:**
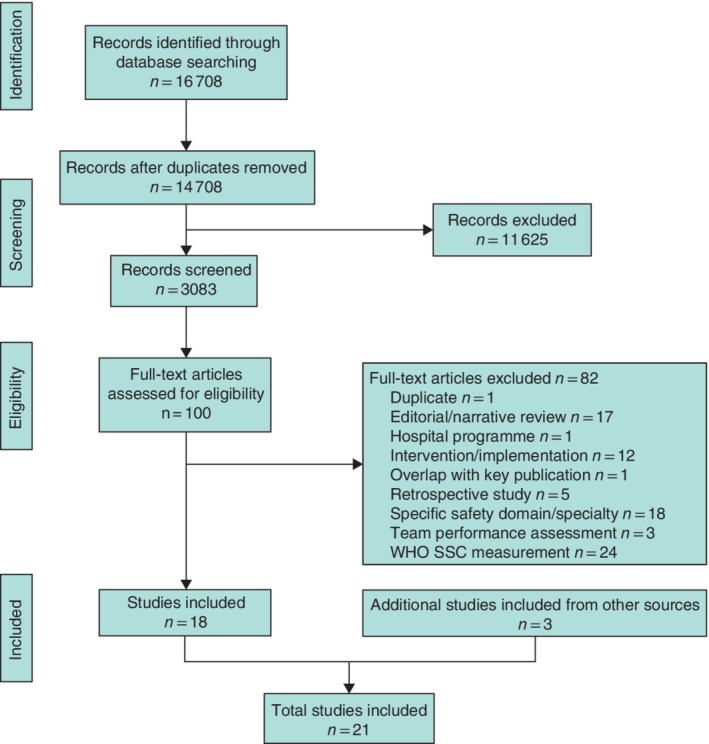
PRISMA diagram for the systematic review SSC, Surgical Safety Checklist.

From 16 708 papers identified in the four databases, 14 708 studies remained after removal of duplicates. Some 100 publications were considered eligible for full‐text screening, and 82 were excluded after further examination. Three additional studies were included, identified by hand‐searching, resulting in the inclusion of 21 studies for data analysis (*Fig*. 1).

### Study characteristics

The key features of the 21 studies are outlined in *Table* *S1* (supporting information). Most studies were conducted in the UK (8 studies) and USA (6). Remaining studies were performed in Austria (1), Belgium (1), Egypt (1), the Netherlands (3) and Switzerland (1). There were 19 cross‐sectional and two prospective cohort studies. Various surgical procedures and perioperative phases were studied, such as patient admissions to surgery wards, operating room and recovery area, and postoperative surgery ward area.

### Study quality

One study^19^ was excluded from further analysis because of low outcome and analysis scores (*Appendix* *S2*, supporting information). Thus, 20 studies^20^, ^21^, ^22^, ^23^, ^24^, ^25^, ^26^, ^27^, ^28^, ^29^, ^30^, ^31^, ^32^, ^33^, ^34^, ^35^, ^36^, ^37^, ^38^, ^39^ showing good internal and external validity remained, and were used for in‐depth analysis.

### Risk‐assessment methods

An overview of the included studies on prospective risk‐assessment methods for identifying perioperative patient safety risks, targeted risk areas, characteristics and feasibility is shown in *Table* 1. Five categories of prospective risk‐assessment methods included: direct AE surveillance (3 studies), direct (in‐person) observation (16), (modified) Healthcare Failure Mode and Effect Analysis (m‐HFMEA™; Department of Veterans Affairs, National Center for Patient Safety, Ann Arbor, Michigan, USA) (5), indirect observation (4) and use of questionnaires (3). In 11 studies a combination of methods was described (*Table* 1). m‐HFMEA™ methods and direct AE surveillance methods were always combined with direct observations. Furthermore, seven studies described the use of one or more additional prospective assessment tools: contextual inquiries (3 studies), interviews (4), photographs (1) and protocol assessment (1) (*Table* 1). Risk assessments were conducted by various professionals, such as surgeons, medical students and independent consultants (*Appendix* *S3*, supporting information).

**Table 1:** Overview of prospective perioperative risk‐assessment methods (20 studies)

	Risk‐assessment method					
Reference	Direct AE surveillance	Direct observation	(Modified) HFMEA™	Indirect observation	Questionnaire	Supplementary prospective tool
Anderson et al.^29^		Yes	Yes			Interviews
Bentz et al.^21^	Yes	Yes				Interviews
Blikkendaal et al.^38^				Yes	Yes	
Borns et al.^35^				Yes		
Catchpole et al.^33^		Yes		Yes		
Christian et al.^23^		Yes				
Davis et al.^36^					Yes	
Gurses et al.^26^		Yes				Contextual inquiries, photographs
Hamilton et al.^22^	Yes	Yes				
Heideveld‐Chevalking et al.^28^		Yes				
Heideveld‐Chevalking et al.^37^		Yes			Yes	Interviews, protocol assessments
Hu et al.^34^				Yes		
Johnston et al.^30^		Yes	Yes			
Kaul and McCulloch^20^	Yes	Yes				Contextual inquiries
Kreckler et al.^24^		Yes				Interviews
Marquet et al.^39^		Yes	Yes			
Nagpal et al.^31^		Yes	Yes			
Parker et al.^25^		Yes				
Smith et al.^32^		Yes	Yes			
Thompson et al.^27^		Yes				Contextual inquiries
Total	3	16	5	4	3	

AE, adverse event; HFMEA, Healthcare Failure Mode and Effect Analysis.

#### *Direct adverse event surveillance*

Three studies used a prospective AE surveillance method, all combined with direct observations. The actual AE rate ranged between 6 and 23 per cent, and 8–20 per cent of AEs were considered preventable^20^, ^21^. Methods included surgeon surveillance, institutionalized monitoring policy of self‐reporting of AEs, direct observations and interviews with perioperative staff members. Recently, direct AE observation was correlated with two retrospective AE reporting systems in 211 surgical cases. Overall, the rate of variance reported by safety observers was 65 per 100 cases, compared with seven per 100 cases for handwritten reporting cards and one per 100 cases using the electronic reporting system. However, the preventability of (potential) AEs was not reported^22^.

#### *Direct (in‐person) observation*

In total, 16 of the 20 studies used direct observations to identify and analyse disruptions that may lead to AEs in surgical care. Ten of these studies combined direct observations with other methods (*Table* 1). Problems in communication and information flow, and workload with competing tasks were found to have a measurable negative impact on team performance and patient safety^23^. In addition, length of stay was significantly associated with (potential) AEs in emergency general surgery admissions^24^. A surgical flow disruption tool to classify flow disruptions in cardiovascular operations has been also proposed^25^, with strong interrater reliability.

Other methods included the combination of direct observation, contextual inquiries and photographs to identify and categorize hazards in cardiac surgery^26^. Hazards were related to care providers (such as practice variations), tasks (such as high workload), tools and technologies (such as poor usability), physical environment (such as cluttered workspace), organization (such as hierarchical culture) and processes (such as non‐compliance with guidelines). A peer‐to‐peer assessment model in cardiovascular operating rooms identified six priority hazard themes including: safety culture, teamwork and communication, prevention of infection, transitions of care, failure to adhere to practices or policies, and operating room layout and equipment^27^. Finally, a Surgical Patient safety Observation Tool (SPOT) was developed and tested to measure and benchmark perioperative patient safety performance^28^. SPOT showed good measurability, applicability and improvement potential for compliance to (inter)national patient safety guidelines. The tool showed good discriminatory capacity, with a range of 72·5–100 per cent in compliance performance between hospitals and departments.

#### *(Modified) Healthcare Failure Mode and Effect Analysis (m‐HFMEA™)*

Five studies used a m‐HFMEA™ method combined with direct observations to identify and prioritize hazards. The m‐HFMEA™ method incorporates a multistage approach that utilizes the expertise of an interprofessional team. This includes the development of process flow charts, hazard scores and decision trees to define areas of potential failure where the patient is most susceptible to avoidable harm. Using this methodology, hazardous failures identified included hand hygiene, isolation of infection, vital signs, medication delivery and handover^29^, as well as communication problems, understaffing and hierarchical barriers^30^. Studies also reported that most failures were identified before surgery^31^. One study^32^ used a structured what‐if technique (SWIFT) to identify non‐operative risks in group sessions. A total of 102 risks were identified, and the top 20 recommendations were judged to encompass about 75 per cent of the total estimated risk attributable to the processes considered^32^.

#### *Indirect observations by video recordings*

Four studies used indirect observations to assess performances and disruptions in surgical procedures, to identify perioperative risk. In one study^33^, a correlation was found between the occurrence of minor problems, intraoperative performance and duration of surgery. Minor problems were defined as those negative events that were seemingly innocuous, and intraoperative performance as the proportion of key operating tasks that were disrupted. In addition, eight major problems – events that compromised directly the safety of the patient or the quality of the treatment – were observed. Interestingly, using a method of audio‐video recording, transcribing ten highly complex operations and then identifying deviations by majority consensus of a multidisciplinary team, a mean of one deviation every 79 min during complex procedures has been reported^34^. Similarly, using videos, a statistically significant correlation between accurate handover and adherence to guidelines was found in an advanced trauma paediatric resuscitation bay^35^.

#### *Questionnaires*

Three studies used questionnaires as a prospective risk assessment method. One paper^36^ reported that women, educated patients and those in employment were more willing to ask questions, whereas men, less educated or unemployed people were less willing to challenge healthcare staff regarding their care than to ask healthcare staff factual questions. However, doctor's instructions to the patient increased patient willingness to challenge doctors and nurses. Some 10 years later, a Self‐assessment Instrument for Perioperative Patient Safety (SIPPS) was developed and validated by perioperative healthcare staff^37^. SIPPS showed good measurability (99·8 per cent) and applicability (99·9 per cent), although mean compliance was 76 per cent among five institutions, and mixed results were shown in discriminatory capacity^37^. In 2018, a Surgical Safety Questionnaire was developed^38^ to be completed after gynaecological procedures, by surgeons, scrub nurses and anaesthetists. The validity of the questionnaire was confirmed by comparison with video analysis. Potential safety concerns were reported, related to surgical flow disturbances consuming time and to using a new instrument or device^38^.

### Quality characteristics and feasibility of included studies

Various quality characteristics were reported in five of the included studies: three direct observation studies^25^, ^26^, ^28^ and two questionnaires^37^, ^38^ (*Appendix* *S3*, supporting information). The direct observation method was reported with a strong interrater reliability^25^. In addition, good measurability, good applicability, good improvement potential and good/mixed results in the discriminatory capacity were reported for direct observation and questionnaire methods^28^, ^37^.

The Surgical Safety Questionnaire validation^38^ resulted in reliable quantitative results, allowing this questionnaire to be considered a validated tool to evaluate and maintain surgical safety, which may help prevent potential safety hazards during minimally invasive procedures.

Feasibility of the studied methods was reported in ten included studies, with quantified results in three studies. Whereas (in)direct observation methods and direct AE surveillance methods were described as simple^20^, ^25^, clear^25^, practical^33^ and easy to use^28^, the (modified) HFMEA™ method was considered time‐consuming^33^, requiring considerable personnel resources^31^.

An overview of prospective perioperative risk‐assessment methods and key characteristics is presented in *Table* 2. Methods were found to detect four risk areas: (potential) AEs and risk factors, problems and errors; perioperative process flow disruptions and hazardous failures within these processes; adherence to evidence‐based guidelines; and individual or team practice performance (disruptions of operational key tasks).

**Table 2:** Overview of prospective perioperative risk‐assessment methods with their targeted risk areas, and reported quality and feasibility characteristics

	Risk assessment method				
Study characteristics	Direct AE surveillance	Direct observation	(Modified) HFMEA	Indirect observation	Questionnaire
**Targeted risk areas**					
(Potential) AEs	x	x		x	
Perioperative process flow disruptions		x	x	x	
Adherence to standard operating procedures		x		x	x
Individual or team performance		x		x	
**Quality characteristics**					
(Face) validity		+			+
Interrater reliability		+			+
Measurability, applicability, improvement potential, discriminatory capacity		+			+
**Feasibility**					
Easy to use	+	+		+	
Clear formulation, relevant, good answering possibility, acceptable time effort					+
Requiring considerable personnel			−		
Time‐consuming		−	−	−	

x, Targeted risk area; +, advantage; −, disadvantage. AE, adverse event; HFMEA, Healthcare Failure Mode and Effect Analysis.

## Discussion

Literature review identified five categories of prospective perioperative risk‐assessment method. Overall, about half of the studies addressed more than one methodology, and m‐HFMEA™ and direct AE surveillance were always combined with direct observations.

At present, the primary prospective risk‐assessment method that may best help to target perioperative hazards is direct (in‐person) observation. This method covers a broad spectrum of perioperative risk areas and is relatively straightforward to perform. Direct observation was used across different phases in the perioperative care process and for various procedures and operation types, especially in high‐risk surgery (such as cardiovascular surgery and gastrointestinal oncology).

In contrast, the method of indirect observation was studied less frequently, although it targeted the same risk areas as direct observation. Indirect observation by video recording allowed accurate and detailed assessment, and provided the opportunity to analyse data more efficiently. Participation in video recordings, however, was sometimes limited, reflecting a prevailing culture of unease about personal video observation^33^. Both types of observation (direct and indirect) are limited by observer variation, and a potential Hawthorne effect (the type of reactivity in which individuals modify an aspect of their behaviour in response to their awareness of being observed) might be stronger during in‐person observations owing to the visibility of the observers.

(Modified) HFMEA™ was found to be helpful in understanding processes and identifying potential hazardous failures in the perioperative process. In all m‐HFMEA™ studies, additional real‐time clinical observation was used, both to help map the process and to confirm assessed failure modes^39^. In addition, direct (real‐time) AE surveillance was always combined with observation methods, and this combination detected tenfold more AEs than common (retrospective) AE reporting systems^22^.

Finally, three different questionnaire studies^36^, ^37^, ^38^ gave insight into the views and perspectives of caregivers and patients. A disadvantage of this method is results being based on just a sample of the study population.

Validity and feasibility of included methods were studied poorly and need further research, although it seems that validation methods were applied more frequently in more recent studies. Various quality characteristics were studied in three studies^25^, ^26^, ^28^ on direct observation and in two questionnaire studies^37^, ^38^, showing satisfactory results. Whereas (in)direct observation methods and direct AE surveillance methods were described as feasible^20^, ^25^, ^28^, ^33^, the (modified) HFMEA™ method was considered time‐consuming^39^, requiring considerable personnel resources^31^. Irrespective of the risk‐assessment method used, involved personnel must be trained in evaluation and analysis to give consistent and meaningful results.

This review offers a comprehensive overview of the availability of prospective methods used for identification and monitoring of perioperative patient safety risks that may lead to AEs, and their advantages and disadvantages.

Intraoperative safety interventions, such as a time‐out procedure, are intended to reduce patient safety risks such as wrong‐site operations. The character of such an intervention is to target directly possible risks and prevent AEs on a single‐patient level. However, the intervention itself is not designed for prospective assessment of performance variability (how a time‐out procedure is performed). More specifically, an AE such as wrong‐site surgery can be detected primarily and thus prevented by a time‐out procedure. However, a retrospective analysis can be used to detect why wrong‐site surgery occurred in a specific or multiple cases, although factors that possibly lead to wrong‐site surgery should be identified with a prospective risk assessment.

The choice of risk‐assessment method can affect the detection rate of AEs up to 50‐fold^40^. Nevertheless, a prospective risk assessment may be performed at each chosen moment, and specific perioperative target areas can also monitor perioperative patient safety intervention effects over time, and enable benchmarking prospectively.

This review has some limitations. First, the terms compliance or adherence were not included in the literature search. However, terms such as guidelines and extensive hand‐searching of the references were used in order not to miss relevant studies. Second, the literature before 1999, when the paper ‘To err is human’ was published by the US Institute of Medicine^41^, was not covered. Third, studies that involved more than one surgical subspecialty and those that targeted specific patient safety issues (such as surgical‐site infection or medication safety) could have been missed. Finally, the designs of included studies were heterogeneous and no single checklist fitted well, such as the COnsolidated criteria for Reporting Qualitative research (COREQ) checklist for qualitative studies^42^ or the checklist for clinimetric criteria for the development and validation of measurement instruments^43^.

The complexity of surgical care, combined with heavy workloads, fatigue and production pressure, makes the surgical care process particularly vulnerable to AEs^44^. At the same time, despite this vulnerability, most surgical procedures are performed proficiently and safely, highlighting the resilience of individuals and surgical teams to the potential adversity of the setting^44^. This suggests that, in addition to studying AEs and errors, it seems crucial also to study the achievements of teams and how threats to safety are managed successfully. Risk assessments should move forward by combining two complementary views of thinking of safety: learning from both how things went wrong, and how things go right^45^.

According to the present findings, a direct observation method is required, ideally in combination with at least one of the following methods: indirect observation; direct AE surveillance; m‐HFMEA™; questionnaires; and supplementary tools such as interviews, contextual inquiries, photographs and protocol assessment. These methods can be used in a complementary manner to one another, each targeting a different aspect of perioperative care^46^. Furthermore, if similar methods are used, benchmarking in hospitals and departments is possible, enabling learning from both low‐ and high‐practice performances.

## Supporting information


**Appendix S1.** Supporting Information
