# Supplementary material for: Prospective methods for identifying perioperative risk‐assessment methods for patient safety over 20 years: a systematic review
Source: BJS Open. 2019 Dec 17;4(2):197–205. doi: 10.1002/bjs5.50246 (PMC7093778; doi:10.1002/bjs5.50246)
Supplement: Supplementary file 1 — Appendix S1. Supporting Information [file BJS5-4-197-s001.docx]

**BJS5_50246**

**Prospective methods for identifying perioperative risk-assessment methods for patient safety over 20 years: a systematic review**

**A. J. Heideveld-Chevalking, H. Calsbeek, J. Hofland, W. J. H. J. Meijerink and A. P. Wolff**

**Appendix S1** Patient–Determinant–Outcome (PDO) search strategy for MEDLINE database

**Patient/target group**

Perioperative Care[Mesh:noexp] OR Intraoperative Care[MeSH] OR Perioperative Nursing[MeSH] OR postoperative Care[MeSH] OR Preoperative Care[Mesh:noexp] OR Perioperative Period[Mesh:noexp] OR Intraoperative Period[MeSH:noexp] OR Postoperative Period[MeSH] OR Preoperative Period[MeSH] OR "Surgical Procedures, Operative/complications"[Mesh] OR Perioperative[Tiab] OR preoperative[Tiab] OR intraoperative[Tiab] OR postoperative[Tiab] OR operative[Tiab] OR surgical[Tiab] OR surgery[Tiab]

AND

**Determinant**Risk Management[Mesh:noexp] OR Risk Assessment[Mesh:noexp] OR Healthcare Failure Mode and Effect Analysis[MeSH] OR Safety Management[Mesh:noexp] OR "Quality Assurance, Health Care"[Mesh:noexp] OR Benchmarking[MeSH] OR Clinical Audit[Mesh:noexp] OR Medical Audit[MeSH] OR Nursing Audit[MeSH] OR Credentialing[MeSH] OR Guidelines as Topic[Mesh:noexp] OR Practice Guidelines as Topic[MeSH] OR Healthcare Time Out[MeSH] OR Total Quality Management[MeSH] OR "Accident Prevention"[Mesh:noexp] OR audit*[Tiab] OR tracer[Tiab] OR tracers[Tiab] OR tool[Tiab] OR tools[Tiab]

AND

**Outcome**"Medical Errors"[Mesh:noexp] OR "Medication Errors"[Mesh:noexp] OR "Inappropriate Prescribing"[Mesh] OR Healthcare Near Miss[MeSH] OR "Patient Harm"[Mesh] OR Patient safety[MeSH] OR adverse event[Title] OR adverse events[Title] OR patient safety[Title] OR harm[Title] OR error[Title] OR errors[Title] OR hazard*[Title] OR incident[Title] OR incidents[Title] OR accident[Title] OR accidents[Title] OR complication*[Ti] OR adverse event[Ot] OR adverse events[Ot] OR patient safety[Ot] OR harm[Ot] OR error[Ot] OR errors[Ot] OR hazard*[Ot] OR incident[Ot] OR incidents[Ot] OR accident[Ot] OR accidents[Ot] OR complication*[Ot]

**Table S1** Key characteristics of the 21 selected studies

| **Authors** | **Study period and Country** | **Aim of the study** | **Study design** | **Perioperative phase / location** | **Target Group/sample size** | **prospective risk measurement methods** |
| --- | --- | --- | --- | --- | --- | --- |
| Anderson et al.^1^ | 2012  UK | (1)To identify health care processes occurring in surgical wards, (2) prioritize hazardous processes, (3) identify hazardous failures within prioritized processes, (4) determine their causes and recommend interventions | Cross-sectional study | Surgical ward processes | 5 general surgery wards in 3 acute hospitals | Modified HFMEA  Direct observations  Interviews |
| Benz et al.^2^ | 2009 Austria | To estimate the risk of having an AE and identify potential preventable AEs and associated risk factors | prospective cohort study | A reproductive surgery endoscopy unit | 796 patients undergoing surgery at a reproductive surgery unit | AE surveillance  Self-reporting of AEs. direct-observations, Interviews |
| Blikkendaal et al.^3^ | 2018 Netherlands | To observe whether surgical teams are capable of measuring surgical safety, especially with regard to the introduction of new techniques and technologies during a series of minimal invasive procedures. | Cross-sectional study | Intraoperative | 40 patients undergoing a laparoscopic hysterectomy procedure | A short questionnaire followed by video observations |
| Borns et al.^4^ | 2018 Switzerland | To analyse the adherence to advanced trauma life support guidelines and identify management errors | Cross-sectional study | Patient admissions (day and night) | 128 patients (66% surgical diseases 34% medical diseases) admitted to a paediatric Resuscitation Bay | Video recordings |
| Catchpole et al.^5^ | 2007  UK | To assess deficiencies in systems, iin order to prevent them from escalating to more serious situations | Cross-sectional study | Starting immediately before the patient was transferred to the OR and ending after patient handoff to post anesthesia/ICU | 24 paediatric cardiac and 18 orthopaedic operations in two hospitals | Direct observations and video recording |
| Christian et al.^6^ | 2006 USA | To identify system features that influence patient safety in the operating room | Cross-sectional study | Preoperative (starting with the pre-anaesthesia holding unit), intraoperative and postoperative phases | 10 complex general surgery cases (colorectal and hepatobiliary) in one hospital | Direct observations |
| Davis et al.^7^ | 2008  UK | To explore patients’ willingness to question health staff about quality and safety of their healthcare | Cross-sectional study | Patients were recruited postoperatively over a 3-month period | a sample of 80 patients who had undergone a number of different surgical procedures from 4 wards in one hospital | patient self-reporting through questionnaire |
| Gurses et al.^8^ | 2012 USA | To identify and categorize hazards in the cardio-vascular OR. | Cross-sectional study | Intraoperative period | 20 cardiac surgeries in 5 hospitals | Direct observation, contextual inquiry and photographs |
| Hamilton et al.^9^ | 2018 USA | To evaluate directly observed variances and to correlate these with the 2 established variance reporting systems | Cross-sectional study | Intraoperative | 211 surgical cases (25%)) in one hospital | Direct observation |
| Heideveld et al.^10^ | 2018 Netherlands | To develop, test and validate a Surgical patient safety Observation Tool (SPOT), in order to monitor, benchmark and improve perioperative patient safety performance | Cross-sectional study | Perioperative – from admission to hospital to discharge | 22 hospitals participated in the first phase of the study, and was pilot tested in 8 hospitals. | Direct observation |
| Heideveld et al.^11^ | 2018 Netherlands | To validate a Self-assessment Instrument for perioperative patient Safety (SIPPS) monitoring and benchmarking compliance to safety standards | Cross-sectional study | Perioperative – from admission to hospital to discharge | 13 hospitals participated in first Delphi round; perioperative professionals pilot tested the tool in 5 hospitals | Self-assessment questionnaire |
| Hu et al.^12^ | 2012 USA | To (1) develop a methodology of audio-video recording operations, and (2) identify the key factors that play a role in the aetiology and/or recovery of unanticipated events in the OR. | Cross-sectional study | From OR set up (the opening of sterile kits) through patient leaving the OR | 10 high-complex operations within general surgery and surgical oncology that had published expected complication rates of >20%. | Video/audio recordings |
| Johnston et al.^13^ | 2015 UK | To systematically risk assess and analyze the escalation of care process to identify problems and provide recommendations to improve patient safety postoperatively on the surgical ward | Cross-sectional study | Postoperative surgery ward area | 6 Surgical wards in 4 London hospitals, | HFMEA and direct observations |
| Kaul et al.^14^ | 2007 UK | To determine the true rate of adverse event in surgical patients by prospective surveillance and independent analysis | Cross-sectional study | Patients admissions to a surgical ward | All routine and emergency admissions to a single surgical ward, during a specified 30-day period, including 113 admissions | prospective surveillance of AEs  contextual inquiries |
| Kreckler et al.^15^ | 2009  UK | To evaluate patient safety using process and outcome measures in parallel. To develop a process-based system for evaluating ward safety. To define the level of noncompliance with safety-related processes associated with a measured frequency of patient harm. | exploratory cohort study | A surgical ward | An emergency surgical unit | Direct observation |
| Marquet et al.^16^ | 2013  Belgium | To use the HMFEA method to evaluate the process flow for ear, nose and throat (ENT) patient, and to redesign the process to enhance patient safety | Cross-sectional study | ENT ward and OR | In 2 One Day Clinics (one large one small), ENT processes | HMFEA and direct observations |
| Nagpal et al.^17^ | 2010  UK | To apply HFMEA to the information transfer and communication process in the surgical journey of patients. | Cross-sectional study | 4 main phases in surgical care: preoperative assessment, pre-procedural teamwork, postoperative handover, and daily ward care | an acute teaching hospital in the UK that is also a tertiary referral centre for major gastrointestinal cancer surgery | HMFEA and direct observations |
| Parker et al.^18^ | 2010 USA | (1)To develop a reliable tool to prospectively categorize surgical flow disruptions and the conditions that predispose a surgical team to adverse events and their impact on patient safety in the OR. (2)To create and validate a practical tool that systematically classified surgical flow disruptions. | Cross-sectional study | From the time of patient entry into the OR until closure of the surgical wound | 12 cardiac operations followed by 10 cardiac surgeries at one Hospital | A developed and validated surgical flow disruption tool (SFDT) , including direct observations |
| Sayed et al.^19^ | 2013 Egypt | To assess the patient safety status in the OR. To identify hazards, and to assess risks that jeopardize safety | Cross-sectional study | Intraoperative – sign in, time-out and sign-out stages | 100 selected patients undergoing general surgical and urological surgical procedures carried out in 3 ORs of a governmental hospital | Interviews, and a risk assessment method |
| Smith et al.^20^ | 2010 UK | (1)To identify prospectively non-operative risks (2) to prioritize recommendations arising by cost, ease and likely speed of implementation. | Cross-sectional study | Perioperative process, from admission to the surgical ward through to the OR and the recovery room | Group sessions totalling 20 clinical and administrative healthcare staff involved in perioperative care and risk experts convened by the UK national patient Safety Agency | Modified HMFEA, direct observation, a customized structured 'what if technique'(SWIFT) |
| Thompson et al. ^21^ | 2015 USA | To develop a scientifically sound and feasible peer-to-peer assessment model that allows health-care organizations to evaluate patient safety in cardiovascular operating rooms and to establish safety priorities for improvement. | Cross-sectional study | Intraoperative – cardiovascular surgical OR | A multidisciplinary team, composed of organizational sociology, organizational psychology, applied social psychology, clinical medicine, human factors engineering, and health services researchers; 5 sites | survey assessments, interviews, direct observations and contextual inquiries |

1. Anderson O, Brodie A, Vincent CA, et al. A systematic proactive risk assessment of hazards in surgical wards: a quantitative study. *Ann Surg* 2012; 255(6):1086-92.

2. Bentz EK, Imhof M, Pateisky N, et al. Clinical outcome monitoring in a reproductive surgery unit: a prospective cohort study in 796 patients. *Fertility and Sterility* 2009; 91(6):2638-2642.

3. Blikkendaal MD, Driessen SRC, Rodrigues SP, et al. Measuring surgical safety during minimally invasive surgical procedures: a validation study. *Surgical Endoscopy* 2018; 32(7):3087-3095.

4. Borns J, Ersch J, Dobrovoljac M, et al. Video Recordings to Analyze Preventable Management Errors in Pediatric Resuscitation Bay. *Pediatr Emerg Care* 2018.

5. Catchpole KR, Giddings AE, Wilkinson M, et al. Improving patient safety by identifying latent failures in successful operations. *Surgery* 2007; 142(1):102-10.

6. Christian CG, M.L; Roth, E.M; Sheridan, T.B; Gandhi, T.K; Dwyer, K; Zinner, M.J; Mierks, M.M. A prospective study of patient safety in the operating room. *Surgery* 2006; 139(2):159-173.

7. Davis RE, Koutantji M, Vincent CA. How willing are patients to question healthcare staff on issues related to the quality and safety of their healthcare? An exploratory study. *Quality & Safety in Health Care* 2008; 17(2):90-96.

8. Gurses AP, Kim G, Martinez EA, et al. Identifying and categorising patient safety hazards in cardiovascular operating rooms using an interdisciplinary approach: a multisite study. *BMJ Qual Saf* 2012; 21(10):810-8.

9. Hamilton EC, Pham DH, Minzenmayer AN, et al. Are we missing the near misses in the OR?-underreporting of safety incidents in pediatric surgery. *J Surg Res* 2018; 221:336-342.

10. Heideveld-Chevalking AJ, Calsbeek H, Emond YJ, et al. Development of the Surgical Patient safety Observation Tool (SPOT). *BJS Open* 2018; 2(3):119-127.

11. Heideveld-Chevalking AJ, Calsbeek H, Griffioen I, et al. Development and validation of a Self-assessment Instrument for Perioperative Patient Safety (SIPPS). *BJS Open* 2018; 2(6):381-391.

12. Hu YY, Arriaga AF, Roth EM, et al. Protecting patients from an unsafe system: The etiology and recovery of intraoperative deviations in care. *Annals of Surgery* 2012; 256(2):203-210.

13. Johnston M, Arora S, Anderson O, et al. Escalation of care in surgery: a systematic risk assessment to prevent avoidable harm in hospitalized patients. *Ann Surg* 2015; 261(5):831-8.

14. Kaul AKM, P.G. Patient Harm in General Surgery-A Prospective Study. *.J Patient Saf* 2007; 3:22-26.

15. Kreckler S, Catchpole KR, New SJ, et al. Quality and safety on an acute surgical ward: An exploratory cohort study of process and outcome. *Annals of Surgery* 2009; 250(6):1035-1040.

16. Marquet K, Claes N, Postelmans T, et al. ENT one day surgery: critical analysis with the HFMEA method. *B-ent* 2013; 9(3):193-200.

17. Nagpal K, Vats A, Ahmed K, et al. A systematic quantitative assessment of risks associated with poor communication in surgical care. *Arch Surg* 2010; 145(6):582-8.

18. Parker SE, Laviana AA, Wadhera RK, et al. Development and evaluation of an observational tool for assessing surgical flow disruptions and their impact on surgical performance. *World J Surg* 2010; 34(2):353-61.

19. Sayed HA, Zayed M, El Qareh NM, et al. Patient safety in the operating room at a governmental hospital. *J Egypt Public Health Assoc* 2013; 88(2):85-9.

20. Smith A, Boult M, Woods I, et al. Promoting patient safety through prospective risk identification: example from peri-operative care. *Qual Saf Health Care* 2010; 19(1):69-73.

21. Thompson DA, Marsteller JA, Pronovost PJ, et al. Locating Errors Through Networked Surveillance: A Multimethod Approach to Peer Assessment, Hazard Identification, and Prioritization of Patient Safety Efforts in Cardiac Surgery. *J Patient Saf* 2015; 11(3):143-51.

**Appendix S2** Quality appraisal of the 21 selected studies (detailed comments available on request)

|  | Section 1  Population | | | | Section 2  Method of selection | | | | Section 3  Outcomes | | | | | | Section 4  Analysis | | | | | Summary validity | |
| --- | --- | --- | --- | --- | --- | --- | --- | --- | --- | --- | --- | --- | --- | --- | --- | --- | --- | --- | --- | --- | --- |
| Study | **1.1** | **1.2** | **1.3** | **Total 1** | **2.1** | **2.2** | **2.3** | **Total 2** | **3.1** | **3.2** | **3.3** | **3.4** | **3.5** | **Total 3** | **4.1** | **4.2** | **4.3** | **4.6** | **Total 4** | **5.1 IV** | **5.2 EV** |
| Anderson et al.^1^ | ++ | ++ | ++ | ++ | ++ | ++ | NA | ++ | ++ | ++ | ++ | NA | NA | ++ | NA | NA | ++ | NA | ++ | **++** | **++** |
| Benz et al.^2^ | ++ | ++ | ++ | ++ | ++ | + | NA | ++ | ++ | ++ | ++ | NA | NA | ++ | ++ | + | + | ++ | + | **++** | **++** |
| Blikkendaal et al^3^ | ++ | + | ++ | ++ | ++ | NA | NA | ++ | ++ | ++ | ++ | NA | NA | ++ | NA | NA | ++ | NA | ++ | **++** | **++** |
| Borns et al.^4^ | ++ | + | ++ | ++ | ++ | + | NA | ++ | ++ | ++ | ++ | NA | NA | ++ | NA | + | ++ | ++ | ++ | **++** | **++** |
| Catchpole et al.^5^ | ++ | + | ++ | ++ | + | + | NA | + | ++ | ++ | ++ | NA | NA | ++ | NR | + | ++ | ++ | ++ | **++** | **++** |
| Christian et al.^6^ | ++ | + | ++ | ++ | + | NA | NA | + | + | ++ | ++ | NA | NA | ++ | NA | NA | ++ | NA | ++ | **++** | **++** |
| Davis et al.^7^ | ++ | + | - | + | + | + | NA | + | ++ | ++ | ++ | NA | NA | ++ | NA | + | ++ | ++ | ++ | **++** | **+** |
| Gurses et al.^8^ | ++ | ++ | NR | + | + | NA | NA | + | + | ++ | ++ | NA | NA | ++ | NA | NA | ++ | NA | ++ | **++** | **+** |
| Hamilton et al.^9^ | + | + | + | + | ++ | NA | NA | ++ | + | ++ | ++ | NA | NA | ++ | NA | NA | + | NA | + | **++** | **+** |
| Heideveld et al.^10^ | + | ++ | ++ | ++ | ++ | NA | NA | ++ | + | ++ | ++ | NA | NA | ++ | NA | NA | + | NA | + | **++** | **++** |
| Heideveld et al.^11^ | + | ++ | ++ | ++ | + | NA | NA | + | ++ | ++ | ++ | NA | NA | ++ | NA | NA | ++ | NA | ++ | **+** | **++** |
| Hu et al.^12^ | ++ | + | + | + | NA | ++ | NA | ++ | ++ | ++ | ++ | NA | NA | ++ | NA | ++ | ++ | ++ | ++ | **++** | **+** |
| Johnston et al.^13^ | ++ | ++ | ++ | ++ | + | ++ | NA | ++ | ++ | ++ | + | NA | NA | ++ | NA | NA | ++ | NA | ++ | **++** | **++** |
| Kaul et al.^14^ | + | + | ++ | + | ++ | + | NA | + | ++ | ++ | ++ | NA | NA | ++ | ++ | ++ | ++ | ++ | ++ | **++** | **+** |
| Kreckler et al.^15^ | ++ | ++ | ++ | ++ | ++ | + | NA | ++ | ++ | ++ | ++ | NA | NA | ++ | NA | ++ | ++ | ++ | ++ | **++** | **++** |
| Marquet et al.^16^ | ++ | + | ++ | ++ | ++ | NA | NA | ++ | + | ++ | ++ | NA | NA | ++ | NA | NA | + | NA | + | **++** | **++** |
| Nagpal et al.^17^ | + | ++ | ++ | ++ | + | NA | NA | + | ++ | ++ | ++ | NA | NA | ++ | NA | NA | ++ | NA | ++ | **++** | **++** |
| Parker et al.^18^ | ++ | + | + | + | + | NA | NA | + | - | ++ | + | NA | NA | ++ | NA | NA | ++ | NA | ++ | **++** | **+** |
| Sayed et al.^19^ | ++ | + | + | + | - | NA | NA | + | - | - | + | NA | NA | - | NA | NA | - | NA | - | **-** | **+** |
| Smith et al.^20^ | + | + | ++ | + | + | NA | NA | ++ | ++ | ++ | ++ | NA | NA | ++ | NA | NA | ++ | NA | ++ | **++** | **+** |
| Thompson et al.^21^ | + | + | + | + | ++ | NA | NA | ++ | ++ | ++ | ++ | NA | NA | ++ | NA | NA | ++ | NA | ++ | **++** | **+** |

**Explanation quality appraisal checklist (ref** [NICE Quality appraisal checklist](https://www.nice.org.uk/process/pmg4/chapter/appendix-g-quality-appraisal-checklist-quantitative-studies-reporting-correlations-and)**)**

| Section 1 Population | 1.1 Is the source population or source area well described?  1.2 Is the eligible population or area representative of the source population or area?  1.3 Do the selected participants or areas represent the eligible population or area? |
| --- | --- |
| Section 2 Method of selection of exposure (or comparison) group | 2.1 Selection of exposure (and comparison) group. How was selection bias minimised?  2.2 Was the selection of explanatory variables based on a sound theoretical basis?  2.3 Was the contamination acceptably low? |
| Section 3 Outcome | 3.1 Were the outcome measures and procedures reliable?  3.2 Were the outcome measurements complete?  3.3 Were all the important outcomes assessed?  3.4 Was there a similar follow-up time in exposure and comparison groups?  3.5 Was follow-up time meaningful? |
| Section 4 analysis | 4.1 Was the study sufficiently powered to detect an intervention effect (if one exists)?  4.2 Were multiple explanatory variables considered in the analyzes?  4.3 Were the analytical methods appropriate?  4.4 Was the precision of association given or calculable? Is association meaningful? |
| Section 5 Summary | 5.1 Are the study results internally valid (i.e. unbiased)?  5.2 Are the findings generalisable to the source population (i.e. externally valid)? |

**Explanation quality appraisal checklist (ref** [NICE Quality appraisal checklist](https://www.nice.org.uk/process/pmg4/chapter/appendix-g-quality-appraisal-checklist-quantitative-studies-reporting-correlations-and)**); quality grading section 1 to 4**

| **++** | Indicates that for that particular aspect of study design, the study has been designed or conducted in such a way as to minimise the risk of bias. |
| --- | --- |
| **+** | Indicates that either the answer to the checklist question is not clear from the way the study is reported, or that the study may not have addressed all potential sources of bias for that particular aspect of study design. |
| **−** | Was reserved for those aspects of the study design in which significant sources of bias may persist. |
| **Not reported (NR)** | Was reserved for those aspects in which the study under review fails to report how they have (or might have) been considered. |
| **Not applicable (NA)** | Was reserved for those study design aspects that were not applicable given the study design under review. |

**Explanation quality appraisal checklist (ref** [NICE Quality appraisal checklist](https://www.nice.org.uk/process/pmg4/chapter/appendix-g-quality-appraisal-checklist-quantitative-studies-reporting-correlations-and)**; overall study quality grading for internal validity (IV) and external validity (EV)**

- ++ All or most of the checklist criteria have been fulfilled, where they have not been fulfilled the conclusions are very unlikely to alter.
- + Some of the checklist criteria have been fulfilled, where they have not been fulfilled, or not adequately described, the conclusions are unlikely to alter.
- – Few or no checklist criteria have been fulfilled and the conclusions are likely or very likely to alter.

References

1. Anderson O, Brodie A, Vincent CA, et al. A systematic proactive risk assessment of hazards in surgical wards: a quantitative study. *Ann Surg* 2012; 255(6):1086-92.

2. Bentz EK, Imhof M, Pateisky N, et al. Clinical outcome monitoring in a reproductive surgery unit: a prospective cohort study in 796 patients. *Fertility and Sterility* 2009; 91(6):2638-2642.

3. Blikkendaal MD, Driessen SRC, Rodrigues SP, et al. Measuring surgical safety during minimally invasive surgical procedures: a validation study. *Surgical Endoscopy* 2018; 32(7):3087-3095.

4. Borns J, Ersch J, Dobrovoljac M, et al. Video Recordings to Analyze Preventable Management Errors in Pediatric Resuscitation Bay. *Pediatr Emerg Care* 2018.

5. Catchpole KR, Giddings AE, Wilkinson M, et al. Improving patient safety by identifying latent failures in successful operations. *Surgery* 2007; 142(1):102-10.

6. Christian CG, M.L; Roth, E.M; Sheridan, T.B; Gandhi, T.K; Dwyer, K; Zinner, M.J; Mierks, M.M. A prospective study of patient safety in the operating room. *Surgery* 2006; 139(2):159-173.

7. Davis RE, Koutantji M, Vincent CA. How willing are patients to question healthcare staff on issues related to the quality and safety of their healthcare? An exploratory study. *Quality & Safety in Health Care* 2008; 17(2):90-96.

8. Gurses AP, Kim G, Martinez EA, et al. Identifying and categorising patient safety hazards in cardiovascular operating rooms using an interdisciplinary approach: a multisite study. *BMJ Qual Saf* 2012; 21(10):810-8.

9. Hamilton EC, Pham DH, Minzenmayer AN, et al. Are we missing the near misses in the OR?-underreporting of safety incidents in pediatric surgery. *J Surg Res* 2018; 221:336-342.

10. Heideveld-Chevalking AJ, Calsbeek H, Emond YJ, et al. Development of the Surgical Patient safety Observation Tool (SPOT). *BJS Open* 2018; 2(3):119-127.

11. Heideveld-Chevalking AJ, Calsbeek H, Griffioen I, et al. Development and validation of a Self-assessment Instrument for Perioperative Patient Safety (SIPPS). *BJS Open* 2018; 2(6):381-391.

12. Hu YY, Arriaga AF, Roth EM, et al. Protecting patients from an unsafe system: The etiology and recovery of intraoperative deviations in care. *Annals of Surgery* 2012; 256(2):203-210.

13. Johnston M, Arora S, Anderson O, et al. Escalation of care in surgery: a systematic risk assessment to prevent avoidable harm in hospitalized patients. *Ann Surg* 2015; 261(5):831-8.

14. Kaul AKM, P.G. Patient Harm in General Surgery-A Prospective Study. *.J Patient Saf* 2007; 3:22-26.

15. Kreckler S, Catchpole KR, New SJ, et al. Quality and safety on an acute surgical ward: An exploratory cohort study of process and outcome. *Annals of Surgery* 2009; 250(6):1035-1040.

16. Marquet K, Claes N, Postelmans T, et al. ENT one day surgery: critical analysis with the HFMEA method. *B-ent* 2013; 9(3):193-200.

17. Nagpal K, Vats A, Ahmed K, et al. A systematic quantitative assessment of risks associated with poor communication in surgical care. *Arch Surg* 2010; 145(6):582-8.

18. Parker SE, Laviana AA, Wadhera RK, et al. Development and evaluation of an observational tool for assessing surgical flow disruptions and their impact on surgical performance. *World J Surg* 2010; 34(2):353-61.

19. Sayed HA, Zayed M, El Qareh NM, et al. Patient safety in the operating room at a governmental hospital. *J Egypt Public Health Assoc* 2013; 88(2):85-9.

20. Smith A, Boult M, Woods I, et al. Promoting patient safety through prospective risk identification: example from peri-operative care. *Qual Saf Health Care* 2010; 19(1):69-73.

21. Thompson DA, Marsteller JA, Pronovost PJ, et al. Locating Errors Through Networked Surveillance: A Multimethod Approach to Peer Assessment, Hazard Identification, and Prioritization of Patient Safety Efforts in Cardiac Surgery. *J Patient Saf* 2015; 11(3):143-51.

**Appendix S3** Results and quality characteristics of the 20 included studies, categorized by main method used

**Table S2 Results and quality characteristics of included studies: direct adverse events surveillance**

| **Author** | **Method description** | **Conducted by** | **Identified risks / focus** | **Key conclusions from authors** | **Reported feasibility** | **Additional reported quality characteristics** |
| --- | --- | --- | --- | --- | --- | --- |
| Benz et al.^1^ | A list of 11 AE categories was compiled of the most often occurring AEs in the experience of the team members. | 2 trained observers monitored the OR and patient ward for AEs on a daily basis. AEs were discussed by the 2 observers and a risk manager, and rated. | Of 60 identified AEs in 45 patients 20% were determined to be preventable | Clinical outcome monitoring is a useful tool for assessing the outcome quality of reproductive surgery by identifying potentially preventable AEs and associated risk factors. | *The AE monitoring system was easily implemented and was well accepted by staff members* | Not reported |
| Hamilton et al.^2^ | Direct observations of near misses and AEs were compared to established handwritten and electronic reporting systems. Observations were analysed and categorized into safety domains and variance categories. | Four medical students were trained to be safety observers to identify near misses and AEs in the OR. One of the authors analysed and categorized these reports. | 211 surgical cases were observed, during which 137 (64%) near misses were identified by direct observation, while 57 (7%) handwritten and 8 (1%) electronic variance were reported. 5 directly observed AEs were not reported in either of the 2 reporting systems. | Despite multiple reporting systems, near misses and AEs remain underreported. Identifying near misses may help address system and process issues before an adverse event occurs. | Not reported | Not reported |
| Kaul et al.^3^ | A clinical observer identified all eligible patients each morning, reviewed their case notes, and questioned the most senior staff member about the expected process and outcome of management for the next 24 hours. The observer recorded actual process and outcome the next morning using information from the admitting team, nursing staff, case notes and patient, repeating this process for each patient until discharge or death. All events were reviewed by a senior author, who also independently assessed a sample of patients (approximately 20%). | An experienced surgeon identified unexpected events and classified them as potentially or actually adverse or not. 3 independent consultant surgeons validated these findings and evaluated the preventability of AEs. | There were 52 (potentially) AEs in 45 patients (40%), of which 29 AEs (23%), of which 8% were considered preventable. | Continuous prospective surveillance revealed an unexpectedly high rate of potential and actual AEs among surgical patients, especially in association with invasive manoeuvres. | *A simple prospective method of recording AEs in real time is feasible* | Not reported |

**Table S3 Results and quality characteristics of included studies: direct observation**

| **Author** | **Method description** | **Conducted by** | **Identified risks / focus** | **Key conclusions from authors** | **Reported feasibility** | **Additional reported quality characteristics** |
| --- | --- | --- | --- | --- | --- | --- |
| Christian et al.^4^ | Minute-to-minute observations were recorded, and later coded and analysed. A qualitative analysis identified major system features that influenced team performance and patient safety. | A team comprised of human factor experts and surgeons | Problems in communication and information flow, workload and competing tasks had measurable negative impact on team performance and patient safety in all 10 cases. 11 events were identified that contributed to or mitigated the overall effect on the patient's outcome. | This study demonstrates the role of prospective observational methods in exposing critical system features that influence patient safety and that can be the targets for patient safety initiatives. | Not reported | Not reported |
| Gurses et al.^5^ | Data were collected and categorized in the LENS study to identify hazards, by direct observations, contextual inquiry and photographs were used to collect hazard data | A multidisciplinary expert panel reviewed the study design and results. Two LENS team observers per case (one clinician and one non-clinician) were drawn from one cardiac anaesthesiologist, one nurse, one human factors engineer and one health services researcher. | 160 hours of observations, and 84 contextual inquiries were recorded. 58 Hazard types included practice variations among care providers, poor teamwork and hierarchical cultures in the COR, violations of guidelines and protocols, and cramped and cluttered workspaces. Most of these hazards have been associated with errors and negative surgical outcomes. | Hazards in cardiac surgery services are omnipresent, indicating numerous opportunities to improve safety. | Not reported | Face validity |
| Heideveld et al.^6^ | 19 perioperative patient safety observation topics were selected from (inter)national guidelines and extracted from locally used observation checklist. After consensus, the final tool SPOT was pilot tested. | A multidisciplinary group of healthcare staff involved in perioperative care | The pilot test showed good measurability and good applicability. The overall patient safety improvement potential with SPOT appeared to be good, and good discriminatory capacity (compliance 72.5-100%) was shown | A comprehensive tool to measure safety of care was developed and validated using a systematic, stepwise method, enabling hospitals to monitor, benchmark and improve perioperative safety performance. | *Respondents considered SPOT easy to use and stated that they were able to use SPOT independently (87,5%)* | Good measurability  Good applicability  Good improvement potential  Good discriminatory capacity |
| Kreckler et al.^7^ | AE, PAE and process measures were studied by direct observation. Each day, clinical staff were interviewed, case notes examined and ward rounds attended to establish plans and expectations for the patients’ course over the next 24 hours. Actual events were reviewed the following day, and any discrepancies with the predicted events were analysed to determine whether or not an AE of PAE had occurred. | Researchers with appropriate clinical experience and understanding were employed to make the observations. Vignettes of all cases were analysed independently by a consultant surgeon, and any classification disagreements resolved by consensus discussion. | Compliance with the 7 processes studied ranged from 23% to 89%. The AE and PAE rates were 11.9% and 13.8% respectively in a 63% sample of admissions (n=607). Length of stay was significantly associated with both AE and PAE (p<0.001). Having an operation was also associated with AE (p=0.001) but not with PAE. | Compliance with individual care processes on a ward with average levels of patient harm is poor. Length of hospital stay increases the risk of both AE and PAE, suggesting a system defect. A bundle of care processes may be useful for monitoring safety improvement. Improvement in safety on acute surgical wards is likely to be best achieved by attention to processed, rather than outcome. | Not reported | Not reported |
| Parker et al.^8^ | A SFDT was developed and tested on their impact on surgical performance. Raters recorded every event that they deemed a surgical flow disruption, | 2 independent raters of different medical and human factor expertise observed 12 cardiovascular operations. After the design period, both raters observed ten surgical cases using SFDT to assess validity and inter-rater reliability | Rating agreement (weighted kappa) for each category across the ten surgeries was moderate to very high, resulting in strong inter-rater reliability for each category on the surgical flow disruption tool. | This research depicts the development and utility of a tool to analyze surgical flow disruptions in the cardiovascular OR with satisfactory inter-rater reliability. This tool is an important first step in systematically categorizing and measuring surgical flow disruptions and their impact on patient safety in the operating room. | *Use of SFDT was simple and clear for observers* | Satisfactory inter-rater reliability |
| Thompson et al.^9^ | Errors were located through networked surveillance. Multiple data collection was involved, such as focused literature review , structured (Telephone) interviews, direct observations and contextual inquiries | A multidisciplinary team, composed of organizational sociology, -psychology, applied social psychology, clinical medicine, human factors engineering, and health services researchers | The top 6 priority hazard themes were as follows: safety culture, teamwork and communication, infection prevention, transitions of care, failure to adhere to practices or policies, and OR layout and equipment. | We integrated the theories and methods of a diverse group of researchers to identify a broad range of hazards and good clinical practices within the cardiovascular surgical OR. Our findings were the basis for a plan to prioritize improvements. These study methods allowed for the comprehensive assessment of a high-risk clinical setting that may translate to other clinical settings. | *This type of evaluation is time intensive and requires many resources to complete* | Not reported |

**Table S4 Results and quality characteristics of included studies: (modified) Healthcare Failure Mode and Effect Analysis**

| **Author** | **Method description** | **Conducted by** | **Identified risks / focus** | **Key conclusions from authors** | **Reported feasibility** | **Additional reported quality characteristics** |
| --- | --- | --- | --- | --- | --- | --- |
| Anderson et al.^10^ | 70 hours of observations recorded all activities in surgical wards. 95 patients and staff quantified hazards associated with 10 health processes. mHFMEA and cause analysis was applied to 5 most hazardous failures . | Two researchers, a surgical scientist and a health care psychologist observed and independently recorded the activities that patients and staff engaged, followed by five multidisciplinary mHFMEA teams. | Hazards associated with 10 health care processes were derived from 81 activities. 5 most hazardous processes included hand hygiene, isolation of infection, vital signs, medication delivery and hand off. Of 190 failures within these processes 50 (26%) were considered hazardous and did not have effective control measures in place. | The study demonstrates how safety in surgical wards can be comprehensively addressed by considering risk associated with all observed activities. | Not reported | Not reported |
| Johnston et al.^11^ | 42 hours of observations formed the basis of an escalations process diagram. A risk-assessment survey identified failures with process steps and attributed hazard scores. Patient safety and clinical risk experts validated hazard scores through a group consensus meeting. Hazardous failures analysed, after which interventions recommended. | 30 surgical staff members and an expert consensus group | Observations identified 33 steps in the escalation process. The risk-assessment survey identified 18 hazardous failures associated with these steps, of which 15 were subjected to cause analysis. Outdated communication technology, understaffing, and hierarchical barriers were identified as root causes of failure. | Failures in the escalation process amenable to intervention were systematically identified. This mapping of the escalation process will allow tailored interventions to enhance surgical training and patient safety. | *The participants felt that the modified HFMEA process was easy to interpret (75%).* | Not reported |
| Marquet et al.^12^ | The process flow for ear, nose and throat patient was prospectively analysed and evaluated by HMFEA to redesign the process to enhance patient safety. Structured real-time clinical observations were conducted to determine whether these were indeed the most important areas for improvement. | Hospital management, ENT physicians, patient safety coordinators and head nurses defined the processes to be analysed. A multidisciplinary HFMEA team developed a process map, conducted hazard analysis and developed improvement actions and outcome measures. | 45 potential failure modes were identified; 25 of them were classified as high or very high risk. Real-time clinical observations confirmed these failure modes. | The HMFEA is a useful instrument for detecting the failure modes in this care process. The involvement of all disciplines and an open safety culture during the procedure were the most important conditions. For the management and the physicians an important conclusion was also that this prospective analysis method, which has international value, could be used in a local context and provide a solid framework for the systematic analysis and prioritization of areas for improvement. | *HFMEA is considered a time consuming method.* | Not reported |
| Nagpal et al.^13^ | A flowchart of the whole surgical process was developed. Potential failure modes were identified and evaluated using a hazard matrix score. Recommendations were determined for certain critical failure modes using a decision tree. | A multidisciplinary team consisting of 4 surgeons, 4 anaesthetists, 6 nurses (ward, OR and recovery), and a psychologist with human factor experience in health care | Most failure modes were identified pre-operatively. 41 of 132 failures were classified as critical, 26 of which were sufficiently covered by current protocols. | Modified HFMEA provided to be a practical approach. Systematic analysis by a multidisciplinary team is a useful method for detecting failure modes. HFMEA method is complementary to quantitative investigations and can identify latent failures in complex surgical care. | *The investigation required a large amount of personnel resources.* | Not reported |
| Smith et al.^14^ | Mapping the perioperative process, developing SWIFT checklist and risk matrix, analyzation and prioritization | Group sessions totalling 20 clinical and administrative healthcare staff involved in perioperative care and risk experts | 102 risks were identified and 95 recommendations made. The top 20 recommendations together were judged to encompass about 75% of the total estimated risk attributable to the processes considered. | This technique can be successfully applied by healthcare staff but expert facilitation of groups is advisable. Such wide-ranging processes can potentially lead to more comprehensive risk reduction than 'single-issue' risk alerts. | Not reported | Not reported |

**Table S5 Results and quality characteristics of included studies: indirect observation**

| **Author** | **Method description** | **Conducted by** | **Identified risks / focus** | **Key conclusions from authors** | **Reported feasibility** | **Additional reported quality characteristics** |
| --- | --- | --- | --- | --- | --- | --- |
| Borns et al.^15^ | Video recording of all patients admitted to the paediatric resuscitation bay was performed. Treatment adherence to advanced trauma life support guidelines and errors per patient was identified. | 2 experienced paediatric emergency physicians reviewed all the videos together and screened and analysed all the patient charts for additional data. | There was a significant correlation (p=0.021)between accurate handover from emergency medical service to hospital physicians and adherence to guidelines (airway, breathing, circulation) and errors related to handover. Some unexpected errors of patient management in the surgical and medical population was revealed by the video recordings. | Video recording is a useful tool to evaluate patient management in the paediatric resuscitation bay. Analyzing errors of missing the adherence to the guidelines helps to pay attention and focus on specific items to improve patient care. | Not reported | Not reported |
| Catchpole et al.^16^ | Operations were classified by risk indicators. Negative events were recorded and organized into 3 levels of clinical importance. The ability of the team working together safety was classified based on a NOTECHS scoring system. Major problems were analysed for trends using *t* tests. | A single observer (a human factors practitioner with experience in observational methods and measurement of human performance) was present during each operation included in the study. Video observations and discussions were used to ensure the quality of the observations. | Operative risk affected intraoperative performance (p=0.004) and duration (p<0.01). 8 major problems were observed showing a strong association with risk, intraoperative performance, teamwork, and the number of minor problems. | Structured observation of effective teamwork in the OR can identify substantive deficiencies in the system, even in otherwise successful operations. Decreasing the number of minor problems can lead to a smoother, safer and shorter operation. Effective teamwork can help decrease the number of small problems and prevent them from escalating to more serious situations. | *This method is a practical way to improve performance* | Not reported |
| Hu et al.^17^ | 10 high-acuity operations representing 44 hours of patient care, we video-recorded and transcribed. Deviations (delays and/or episodes of decreased patient safety) were identified by majority consensus of a multidisciplinary team | 2 surgical research fellows independently generated transcripts of the videos, which were reviewed by a surgeon, a cognitive psychologist and an educational psychologist. This core research team identified deviations by consensus. 3 clinical domain experts (2 surgeons, 1 anaesthesiologist and 1 OR nurse independently categorized these deviations | 33 deviations (10 delays, 17 safety compromises, 6 both) occurred with a mean of one every 79 minutes. Mediation of safety compromises was most frequently accomplished with vigilance, leadership, communication and/or coordination. Nearly all of these deviations (97%) were salvaged by providers. | While recognized in other high risk domains, such human resilience has not yet been described in surgery and has major implications for the design of safety interventions. Systems must be build that simultaneously help avert deviations and train providers to anticipate and deal with those that are unavoidable. | Not reported | Not reported |

**Table S6 Results and quality characteristics of included studies: questionnaire**

| **Author** | **Method description** | **Conducted by** | **Identified risks /focus** | **Key conclusions from authors** | **Reported feasibility** | **Additional reported quality characteristics** |
| --- | --- | --- | --- | --- | --- | --- |
| Blikkendaal et al.^18^ | a Surgical Safety Questionnaire was developed that had to be filled out during 40 laparoscopic hysterectomy procedures, | Surgeons, scrub nurses and anaesthesiologists filled in a short questionnaire direct postoperative | Surgical flow disturbances, equipment/instrument related, environmental related, personnel-related of procedure-related. | The Surgical Safety Questionnaire can act as a validated tool to evaluate and maintain surgical safety during minimally invasive procedures, especially during the introduction of a new intervention. | Not reported | Inter-observer reliability |
| Davis et al.^19^ | A patient self-report survey was developed, comprising 28 questions which assessed patients’ willingness to ask healthcare staff questions that current safety initiatives advise patients to ask. | A researcher went through all the questions with the patient | Surgical patients, particularly those who are men, less educated or unemployed are less willing to challenge healthcare staff regarding their care than to ask healthcare staff factual questions (p<0.001). Doctor's instructions to the patient increased patient willingness to challenge doctors and nurses(p<0.001). Women, educated patients, and patients in employment, were more willing to ask questions (p<0.05) | Surgical patients, particularly those who are men, less educated or unemployed are less willing to challenge healthcare staff regarding their care than to ask healthcare staff factual questions. | Not reported | Not reported |
| Heideveld et al.^20^ | A self-assessment instrument for measuring perioperative patient safety (SIPPS) compliance, meeting international standards, was developed and validated. | Healthcare staff involved in perioperative care | The pilot test showed good measurability and applicability (resp. 99.8 and 99.9%). Room for improvement in perioperative patient safety compliance was demonstrated for all 5 hospitals (mean 76%) | With SIPPS, improvement areas for perioperative patient safety and best practices across hospitals could be identified. | *SIPPS showed good feasibility on four quality criteria (mean 94%): clear formulation (93%), relevancy (92%, good answering possibility (96%) and acceptable time-effort (96%)* | Good measurability  Good applicability  Good improvement potential  Mixed results on discriminatory capacity |

**REFERENCES**

1. Bentz EK, Imhof M, Pateisky N, et al. Clinical outcome monitoring in a reproductive surgery unit: a prospective cohort study in 796 patients. *Fertility and Sterility* 2009; 91(6):2638-2642.

2. Hamilton EC, Pham DH, Minzenmayer AN, et al. Are we missing the near misses in the OR?-underreporting of safety incidents in pediatric surgery. *J Surg Res* 2018; 221:336-342.

3. Kaul AKM, P.G. Patient Harm in General Surgery-A Prospective Study. *.J Patient Saf* 2007; 3:22-26.

4. Christian CG, M.L; Roth, E.M; Sheridan, T.B; Gandhi, T.K; Dwyer, K; Zinner, M.J; Mierks, M.M. A prospective study of patient safety in the operating room. *Surgery* 2006; 139(2):159-173.

5. Gurses AP, Kim G, Martinez EA, et al. Identifying and categorising patient safety hazards in cardiovascular operating rooms using an interdisciplinary approach: a multisite study. *BMJ Qual Saf* 2012; 21(10):810-8.

6. Heideveld-Chevalking AJ, Calsbeek H, Emond YJ, et al. Development of the Surgical Patient safety Observation Tool (SPOT). *BJS Open* 2018; 2(3):119-127.

7. Kreckler S, Catchpole KR, New SJ, et al. Quality and safety on an acute surgical ward: An exploratory cohort study of process and outcome. *Annals of Surgery* 2009; 250(6):1035-1040.

8. Parker SE, Laviana AA, Wadhera RK, et al. Development and evaluation of an observational tool for assessing surgical flow disruptions and their impact on surgical performance. *World J Surg* 2010; 34(2):353-61.

9. Thompson DA, Marsteller JA, Pronovost PJ, et al. Locating Errors Through Networked Surveillance: A Multimethod Approach to Peer Assessment, Hazard Identification, and Prioritization of Patient Safety Efforts in Cardiac Surgery. *J Patient Saf* 2015; 11(3):143-51.

10. Anderson O, Brodie A, Vincent CA, et al. A systematic proactive risk assessment of hazards in surgical wards: a quantitative study. *Ann Surg* 2012; 255(6):1086-92.

11. Johnston M, Arora S, Anderson O, et al. Escalation of care in surgery: a systematic risk assessment to prevent avoidable harm in hospitalized patients. *Ann Surg* 2015; 261(5):831-8.

12. Marquet K, Claes N, Postelmans T, et al. ENT one day surgery: critical analysis with the HFMEA method. *B-ent* 2013; 9(3):193-200.

13. Nagpal K, Vats A, Ahmed K, et al. A systematic quantitative assessment of risks associated with poor communication in surgical care. *Arch Surg* 2010; 145(6):582-8.

14. Smith A, Boult M, Woods I, et al. Promoting patient safety through prospective risk identification: example from peri-operative care. *Qual Saf Health Care* 2010; 19(1):69-73.

15. Borns J, Ersch J, Dobrovoljac M, et al. Video Recordings to Analyze Preventable Management Errors in Pediatric Resuscitation Bay. *Pediatr Emerg Care* 2018.

16. Catchpole KR, Giddings AE, Wilkinson M, et al. Improving patient safety by identifying latent failures in successful operations. *Surgery* 2007; 142(1):102-10.

17. Hu YY, Arriaga AF, Roth EM, et al. Protecting patients from an unsafe system: The etiology and recovery of intraoperative deviations in care. *Annals of Surgery* 2012; 256(2):203-210.

18. Blikkendaal MD, Driessen SRC, Rodrigues SP, et al. Measuring surgical safety during minimally invasive surgical procedures: a validation study. *Surgical Endoscopy* 2018; 32(7):3087-3095.

19. Davis RE, Koutantji M, Vincent CA. How willing are patients to question healthcare staff on issues related to the quality and safety of their healthcare? An exploratory study. *Quality & Safety in Health Care* 2008; 17(2):90-96.

20. Heideveld-Chevalking AJ, Calsbeek H, Griffioen I, et al. Development and validation of a Self-assessment Instrument for Perioperative Patient Safety (SIPPS). *BJS Open* 2018; 2(6):381-391.
